# Nivolumab in combination with cabozantinib for metastatic triple-negative breast cancer: a phase II and biomarker study

**DOI:** 10.1038/s41523-021-00287-9

**Published:** 2021-08-25

**Authors:** Romualdo Barroso-Sousa, Tanya E. Keenan, Tianyu Li, Nabihah Tayob, Lorenzo Trippa, Ricardo G. Pastorello, Edward T. Richardson III, Deborah Dillon, Zohreh Amoozgar, Beth Overmoyer, Stuart J. Schnitt, Eric P. Winer, Elizabeth A. Mittendorf, Eliezer Van Allen, Dan G. Duda, Sara M. Tolaney

**Affiliations:** 1 Medical Oncology, Dana-Farber Cancer Institute, Boston, MA USA; 2 Breast Oncology Program, Dana-Farber/Brigham and Women’s Cancer Center, Boston, MA USA; 3 Oncology Center, Hospital Sírio-Libanês, Brasilia, Brazil; 4 Biostatistics, Dana-Farber Cancer Institute, Boston, MA USA; 5 Broad Institute of Massachusetts Institute of Technology and Harvard, Cambridge, MA USA; 6 Pathology, Brigham and Women’s Hospital, Boston, MA USA; 7 Steele Laboratories for Tumor Biology, Massachusetts General Hospital, Boston, MA USA; 8 Division of Breast Surgery, Department of Surgery, Brigham and Women’s Hospital, Boston, MA USA

**Keywords:** Breast cancer, Breast cancer

## Abstract

This single-arm phase II study investigated the efficacy and safety of cabozantinib combined with nivolumab in metastatic triple-negative breast cancer (mTNBC). The primary endpoint was objective response rate (ORR) by RECIST 1.1. Biopsies at baseline and after cycle 1 were analyzed for tumor-infiltrating lymphocytes (TILs), PD-L1, and whole-exome and transcriptome sequencing. Only 1/18 patients achieved a partial response (ORR 6%), and the trial was stopped early. Toxicity led to cabozantinib dose reduction in 50% of patients. One patient had a PD-L1-positive tumor, and three patients had TILs > 10%. The responding patient had a PD-L1-negative tumor with low tumor mutational burden but high TILs and enriched immune gene expression. High pretreatment levels of plasma immunosuppressive cytokines, chemokines, and immune checkpoint molecules were associated with rapid progression. Although this study did not meet its primary endpoint, immunostaining, genomic, and proteomic studies indicated a high degree of tumor immunosuppression in this mTNBC cohort.

## Introduction

Triple-negative breast cancer (TNBC) accounts for roughly 15% of invasive breast cancer cases^1^. Compared to hormone receptor (HR)-positive and human epidermal growth factor receptor 2 (HER2)-positive breast cancer, TNBC presents a greater risk of distant recurrence within 3–5 years of diagnosis and has worse 5-year survival outcomes^2–4^. Due to the lack of targeted therapies, TNBC is typically treated with systemic chemotherapy^5^. Notably, TNBC can be richly lymphocyte infiltrated, and tumors with high tumor-infiltrating lymphocytes (TILs) have improved prognosis and higher rates of pathologic complete response following chemotherapy^6^.

Recent efforts to develop new therapies for metastatic TNBC (mTNBC) have focused on directly modulating and enhancing immune cell function, particularly with the clinical development of PD-1/PD-L1 inhibitors^7,8^. However, the efficacy of such agents in mTNBC has only been modest when administered as monotherapy^9^. More recently, while the IMpassion-130^10^ and KEYNOTE-355^11^ trials showed improvement in progression-free survival (PFS) with the addition of PD-1/PD-L1 inhibitors to chemotherapy, the IMpassion-131 trial failed to demonstrate superiority of atezolizumab plus paclitaxel versus paclitaxel alone. Additionally, the IMpassion-130 study demonstrated a clinically significant improvement in overall survival and established the combination of atezolizumab plus nab-paclitaxel as the new standard first-line therapy for PD-L1-positive mTNBC^12^.

Yet, most patients with TNBC are not candidates for immunotherapy, and many eligible patients do not derive benefit from it. In fact, several groups have shown that, compared with primary tumors, metastatic breast cancers are “immunologically” cold tumors, phenotypically characterized by low TILs and a marked reduction of interferon gamma^13,14^, which may contribute to the limited benefit of immunotherapy in this setting. Therefore, new therapeutic strategies that increase effector lymphocyte infiltration and reduce intratumoral immunosuppression are required to enhance the efficacy of immune checkpoint inhibitors in mTNBC.

One mechanism that may contribute to immunosuppression in mTNBC is VEGF, a key molecule in tumor angiogenesis. High levels of circulating VEGF and its receptors indicate a poor prognosis for breast cancer patients^15^, and VEGF has been identified as a critical mediator of tumor immunosuppression, by increasing the expression of immune checkpoints that mediate effector CD8+ T-cell exhaustion^16–19^. Thus, targeting the VEGF pathway may be one strategy to increase the efficacy of T-cell-directed immunotherapy, including immune checkpoint inhibitors, and this strategy has now been validated in other advanced cancers^20–23^. Bevacizumab, a monoclonal antibody against circulating VEGF, has been studied in metastatic breast cancer, and while it achieved an initial FDA approval in 2008 based on a significant improvement in PFS when added to paclitaxel, this decision was reversed in 2010 as it was felt the risks did not outweigh the small benefits seen with its addition^24,25^. In addition to VEGF, the MET receptor tyrosine kinase may also contribute to an immunosuppressive tumor microenvironment in mTNBC. MET activation has been linked to increased PD-L1 expression^26,27^, and MET overexpression is associated with poor prognosis in TNBC^28^.

Cabozantinib is an oral small molecule inhibitor of VEGFR2 and MET among other tyrosine kinases^29^, and is FDA-approved for several indications: medullary thyroid cancer^30^, renal cell carcinoma (RCC)^31^, and advanced hepatocellular carcinoma (HCC)^32^. We previously conducted a phase II trial of cabozantinib in mTNBC, which demonstrated a clinical benefit rate of 34%^33^. However, the objective response rate (ORR) was only 9%, indicating limited single-agent activity of cabozantinib^33^. Interestingly, this study demonstrated that cabozantinib treatment was associated with increased circulating CD8+ T cells and decreased CD14+ monocytes^33^. Preclinical studies have demonstrated that cabozantinib can reprogram the tumor immune microenvironment, increase T-cell infiltration in tumors, and synergize with immunotherapy to inhibit tumor growth^34,35^. Moreover, a study evaluating cabozantinib with nivolumab, with or without additional ipilimumab, in patients with genitourinary tumors showed encouraging clinical benefit with manageable toxicity^36^. A subsequent study investigating cabozantinib and atezolizumab in castration-resistant prostate cancer demonstrated an ORR of 32% and a median response duration of 8.3 months^37^. Based on these prior findings, in the present study, we evaluated the safety and efficacy of cabozantinib combined with nivolumab in mTNBC patients previously treated with 0–3 lines of chemotherapy. We also explored molecular mediators of response to this combination regimen using immunohistochemical, genomic, and proteomic studies.

## Results

### Patient characteristics

From 12/15/2017 to 1/24/2019, 18 patients were enrolled into the first stage of the trial. Baseline clinical characteristics are displayed in Table 1. The median age was 58 years (range 41–71), and all patients had an ECOG performance status of 0. The most common sites of metastatic disease were lymph nodes (61%), bone (44%), liver (39%), and chest wall (39%). The median number of prior cytotoxic therapies for mTNBC was 1 (range 0–3). A total of 16 (83%) patients had tumors with 0% ER expression, and 2 patients had tumors with <10% ER expression.

**Table 1:** Baseline characteristics.

Characteristic	Number of patients (%)
Age, years	
Median (range)	58 (41–71)
Sex	
Female	18 (100)
Race	
White	15 (83)
Black	1 (6)
Other	2 (11)
ECOG PS at baseline	
0	18 (100)
Sites of disease	
Lung pleural	6 (33)
Liver	7 (39)
Bone	8 (44)
Breast/chest wall	7 (39)
Lymph node	11 (61)
Soft tissue	3 (17)
Adjuvant or neoadjuvant treatment	
Adjuvant or neoadjuvant hormonal therapy	1 (6)
Adjuvant or or neoadjuvant anthracycline	12 (67)
Adjuvant or neoadjuvant taxane	12 (67)
Lines of chemotherapy for metastasis or recurrence	
Median (range)	1 (0–3)
0	5 (28)
1	6 (33)
2	5 (28)
3	2 (11)
Prior metastatic chemotherapy	
Anthracycline	4 (22)
Taxane	5 (28)
Platinum	11 (61)
Capecitabine	2 (11)
Eribulin	2 (11)
Other chemotherapy	6 (33)
ER, PR, and HER2 status at study entry	
ER and/or PR-positive (≤10%), HER2-negative	2 (11)
ER and PR negative, HER2-negative	16 (89)
PD-L1-positive cells^a^	1 (7)
TILs in metastatic samples^b^	3 (27)

*ECOG PS* Eastern Cooperative Oncology Group Performance Status, *ER* estrogen receptor, *HER2* human epidermal growth factor receptor 2, *PR* progesterone receptor, *TIL* tumor-infiltrating lymphocytes.

^a^PD-L1 was successfully evaluated in 15 patients (8 primary and 7 metastatic samples).

^b^TILs analysis was available in metastatic samples from 11 patients.

### Efficacy

**Fig. 1:**
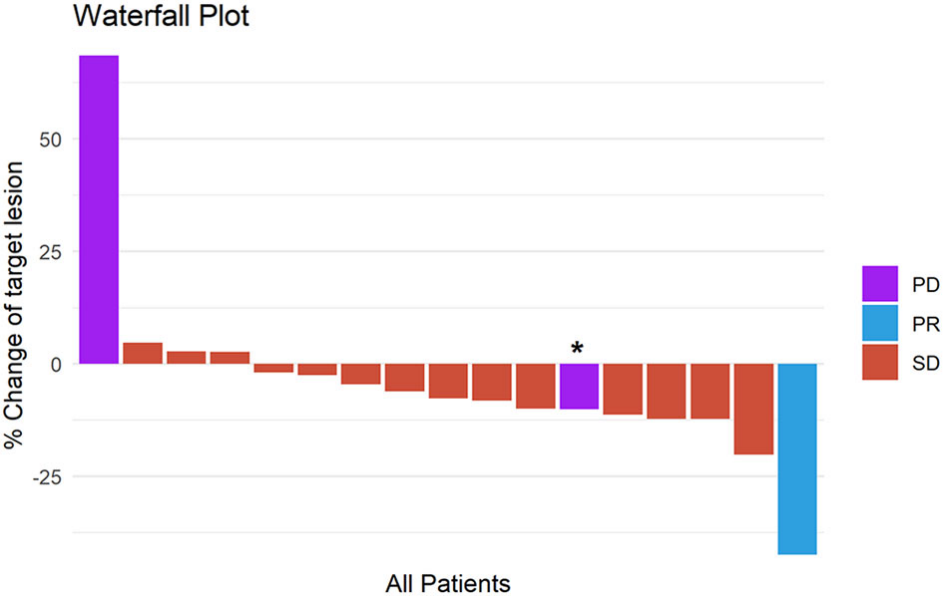
Waterfall plot for best response in metastatic TNBC patients treated with cabozantinib and nivolumab. *Subject 15 had a 10% decrease in the target lesion. However, she had a new lesion. Therefore, her response was classified as PD.

**Fig. 2:**
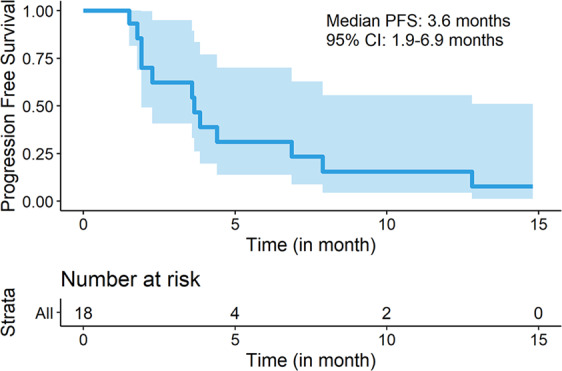
PFS for the included population are shown in months. Kaplan–Meier distributions for progression-free survival in metastatic TNBC patients treated with cabozantinib and nivolumab.

Table 2 shows the best responses to treatment by RECIST 1.1. Only one of the first 18 patients had a partial response (ORR 6%, 95% CI: 0–27); the study therefore did not meet the prespecified criteria to proceed to the second stage of the trial, and it was closed to further accrual. Fourteen patients (78%) had stable disease and two (11%) had progressive disease as the best response (Fig. 1). The clinical benefit rate was 17% (95% CI: 4–41%). Median PFS was 3.6 months (95% CI: 1.9–6.9) (Fig. 2). The single patient with a partial response had an ongoing durable response lasting over 2 years and remained on nivolumab alone at the time of data cutoff on 8/16/2019.

**Table 2:** Best response by RECIST 1.1.

Response	Number of patients (%)
Confirmed PR	1 (6)
SD	14 (78)
PD	2 (11)
Not evaluable	1 (6)
CBR (CR + PR + SD ≥ 24 weeks)	3 (17)

*CBR* clinical benefit rate = CR + PR + SD ≥ 24 weeks, *CR* complete response, *PR* partial response, *SD* stable disease.

### Safety

The combination of nivolumab with cabozantinib was not associated with any unexpected adverse events. All-cause adverse events of any grade occurred in 100% of patients, whereas grade 3–4 adverse events occurred in 83% of patients (Table 3). The most frequent adverse events of any grade were increased aspartate aminotransferase in 50% of patients and palmar-plantar erythrodysesthesia, anorexia, fatigue, hypothyroidism, and increased alanine aminotransferase, each in 39% of patients. The most common grade 3–4 adverse events were palmar-plantar erythrodysesthesia, back pain, and increased aspartate aminotransferase, each in 17% of patients, as well as thromboembolic events, hypertension, fatigue, and increased alanine aminotransferase, each in 11% of patients. Adverse events leading to cabozantinib dose holding occurred in 17 (94.4%) patients; nivolumab was held due to adverse events in 5 (27.8%) patients. Additionally, cabozantinib needed to be dose reduced in 9 patients and permanently discontinued due to toxicity in five patients (Supplementary Table 1).

**Table 3:** All-cause adverse events^a^.

Adverse event	Any grade		Grade 3–4	
	*n*	%	*n*	%
Adverse event of any relatedness	18	100	15	83
AST increased	9	50	3	17
ALT increased	7	39	2	11
Anorexia	7	39	–	–
Fatigue	7	39	2	11
Hypothyroidism	7	39	–	–
Palmar-plantar erythrodysesthesia	7	39	3	17
Myalgia	6	33	–	–
Back pain	5	28	3	17
Diarrhea	5	28	–	–
Hypertension	5	28	2	11
Cough	4	22	–	–
Dysgeusia	4	22	–	–
Dyspnea	4	22	1	6
Edema (limbs)	4	22	–	–
Neutropenia	4	22	–	–
Voice alteration	4	22	–	–
Dry mouth	3	17	–	–
Hyperthyroidism	3	17	–	–
Mucositis (oral)	3	17	–	–
Thromboembolic event	3	17	2	11

*ALT* alanine transaminase, *AST* aspartate aminotransferase.

^a^All events that occurred in ≥15% of study participants.

### Molecular studies

PD-L1 was successfully evaluated in 15 patients (8 primary and 7 metastatic samples) (Table 1). Only one patient had a PD-L1-positive tumor. The test was performed on the primary tumor, and this patient had stable disease as best response. TILs were successfully evaluated in 16 patients (5 primary and 11 metastatic samples). The median TIL level was 10% (range 1–50) in primary samples and 5% (range 1–50) in metastatic samples. Three metastatic samples had TILs > 10, one from the sole patient with a partial response and two from patients with best responses of stable disease, including one with a clinical benefit rate (CBR) (Table 1).

Among the 14 patients (78%) who underwent targeted panel sequencing, no somatic nucleotide variant or copy number alteration was associated with PFS (Supplementary Fig. 1; Supplementary Data 1). The most frequently mutated gene was *TP53* in 86% of sequenced tumors. Six tumors had alterations in *PTEN*, defined as nonsynonymous mutations and/or copy number loss (Supplementary Fig. 1; Supplementary Data 1). *PTEN* alterations were not associated with PFS (Supplementary Fig. 2a; Supplementary Data 1). Three tumors had nonsynonymous mutations in *PIK3CA* (two H1047R missense alterations and one in-frame deletion G106_E109del) (Supplementary Fig. 1; Supplementary Data 1). *PIK3CA* mutations were also not associated with PFS (Supplementary Fig. 2b; Supplementary Data 1). Immunotherapy-related genes had few mutations overall and no copy number alterations associated with PFS (Supplementary Fig. 3; Supplementary Data 1). Median TMB was 4.7 mutations/Mb (range 2.3–10.6), and the single patient with TMB > 10 had progressive disease as the best response to treatment.

**Fig. 3:**
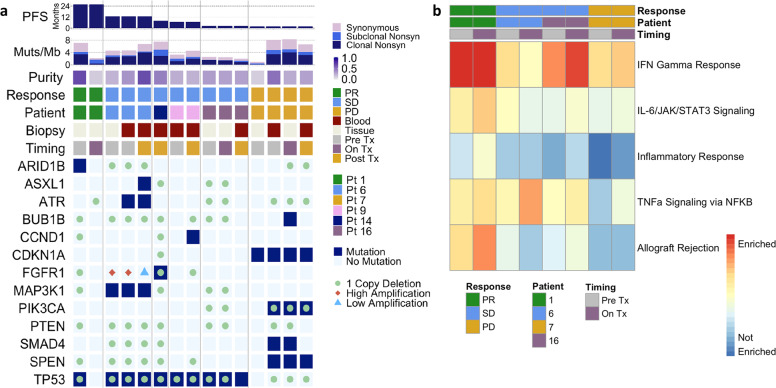
Genomic and transcriptomic analyses of tissues from metastatic TNBC to detect associations with durable responses after nivolumab with cabozantinib. **a** Whole-exome sequencing breast cancer gene alterations in baseline, on-treatment, and post-treatment tissue and blood biopsies from six patients showed no clear association with response: each column represents a tumor or blood biopsy with samples grouped by patient ordered from longest to shortest progression-free survival. **b** Hallmark geneset enrichment analyses of gene expression data revealed immune geneset enrichment in patient with durable response that increased on-treatment, as shown in first two columns. *Muts/Mb* mutations per megabase, *Nonsyn* nonsynonymous*, PD* progressive disease, *PR* partial response, *Pt* patient*, SD* stable disease, *Tx* treatment.

Whole-exome sequencing of tumor and plasma samples revealed similar results as the targeted panel sequencing with no alteration clearly associated with PFS (Fig. 3a, Supplementary Fig. 4, Supplementary Data 1). As expected, low tumor purity samples showed fewer alterations, specifically the on-treatment tumor biopsy of the patient with partial response and the baseline tumor biopsy of the patient with progressive disease (Fig. 3a, Supplementary Fig. 4; Supplementary Data 1). Blood biopsies compared to tissue biopsies at the same timepoint showed additional alterations, for instance, the *ATR* alteration present in the pretreatment blood but not the tumor biopsy sample of patient 6 (Fig. 3a; Supplementary Data 1), consistent with prior studies finding that blood biopsies better capture tumor heterogeneity across multiple sites^38^. To elucidate therapy resistance, post-treatment biopsies were compared to baseline biopsies. Some post-treatment biopsies exhibited mutations not detected in the pretreatment tumor, such as the *ASXL1* alteration in patient 6 and the *CCND1* alteration in patient 14 (Fig. 3a, Supplementary Data 1). In other post-treatment biopsies, we did not observe mutations that were detected in baseline biopsies, such as the *SMAD4* alteration in patient 7, perhaps related to inadequate tumor sampling as suggested by the lower tumor purity of the post-treatment blood sample compared to the baseline blood sample (Fig. 3a, Supplementary Data 1). Mutation clustering analyses indicated that the cancer cell fraction of canonical breast cancer gene mutations, including *TP53*, *ARID1B*, and *FGFR3*, decreased with treatment in tumor biopsies from the partial response patient (Supplementary Fig. 5; Supplementary Data 1), consistent with oncogenic tumor clone regression.

Geneset enrichment analyses revealed higher immune geneset expression in baseline and on-treatment tumor biopsies from the partial response patient (Fig. 3b), specifically the allograft rejection, TNF-α, inflammatory response, IL-6/JAK/STAT3 signaling, and interferon gamma (IFN-γ) response pathways of the cancer hallmark genesets^39^. Geneset enrichment analyses comparing two responding patients (durable partial response and stable disease > 6 months) to two nonresponding patients (progressive disease and stable disease < 6 months) found that these five immune-related genesets were the top five genesets enriched in responders at baseline (FDR q < 0.05, Supplementary Table 2) and that four of these five immune-related genesets were enriched in responders on-treatment (FDR q < 0.01, Supplementary Table 3). Additional geneset enrichment analyses of the tumor biopsies from these four patients showed that the same five immune-related genesets were upregulated in on-treatment compared to baseline samples (FDR q < 0.01, Supplementary Table 4). Interestingly, low tumor purity did not always correlate with increased immune pathway expression as demonstrated by the low purity baseline biopsy sample of the progressive disease patient 7 (Fig. 3a-b). Immune cell deconvolution from RNA sequencing data revealed no significant differences in TILs or absolute immune infiltrate by response category in baseline and on-treatment biopsies (Supplementary Fig. 6; Supplementary Data 1), as well as no consistent change with treatment in either response group (Supplementary Fig. 7; Supplementary Data 1).

Research blood samples were evaluated for circulating levels of angiogenic and inflammatory molecules previously identified as correlates of response to anti-VEGF therapy in TNBC^33^. As expected with anti-VEGFR2 tyrosine kinase inhibitors, treatment was associated with increased plasma VEGF and PlGF and decreased sVEGFR2 concentrations (Supplementary Data 1). Interestingly, combined treatment was associated with substantial and sustained increases in plasma PD-1 concentration in all patients, but no changes in PD-L1 levels. Moreover, treatment was linked to increased plasma CA-IX, IFN-γ, CRTAM, ADA, CXCL9, CXCL10, GZMH, FASL, and HO-1 and reduced plasma IL-12 and TNFRSF12A, but did not affect plasma PD-L1. High baseline plasma levels of multiple markers, including growth factors (VEGF-A, HGF, PlGF, and ANG2), chemokines (CX3CL1, MCP1, MCP2, MCP3, MCP4, CCL4, CCL19, CXCL9, and CXCL10), cytokines (IL-5, IL-6, IL-10, and TNF-α), and immune checkpoint molecules (LAG3, CD27, and CD70) were all associated with shorter PFS (Supplementary Data 1). A longer PFS was associated only with a greater decrease in circulating sVEGFR2 and TNFRSF12A levels between baseline and cycle 2 (Supplementary Data 1).

## Discussion

Combining VEGF inhibitors with immune checkpoint inhibitors is an emerging and validated strategy to enhance response in advanced non-small cell lung carcinoma, HCC and RCC^20–23^. This includes the recent success of cabozantinib with nivolumab versus sunitinib (doubled PFS) in first-line treatment for advanced RCC in the randomized phase III CheckMate 9ER trial (NCT03141177), as well as an ongoing phase III trial of cabozantinib with atezolizumab in advanced HCC (COSMIC-312 study, NCT03755791).

In the present study, we investigated the efficacy and safety of the multi-kinase inhibitor cabozantinib combined with the anti-PD-1 antibody nivolumab in patients with mTNBC. The first stage of the study yielded an ORR of 6% among 18 patients; the study did not meet criteria to proceed to the second stage and therefore was closed early. There were no unexpected or grade 5 adverse events. However, cabozantinib dose reductions and discontinuations were frequent.

We previously reported that cabozantinib increased the fraction of circulating CD8+ T cells and NK cells but that these changes did not correlate with clinical benefit in mTNBC patients^33^. Here, we found that combination of cabozantinib with PD-1 blockade was insufficient to activate anti-tumor immunity in all but one patient.

Tumor molecular analyses indicated that the lack of observed benefit may have been related to the high degree of tumor immunosuppression in the study population: only one patient had PD-L1 breast cancer and few patients had high TILs (3/11). Interestingly, among the 3 with baseline metastatic TILs > 10%, one developed a durable and sustained partial response and another had clinical benefit (stable disease > 24 weeks). In early stage TNBC, the association of TILs and prognosis^6^ as well as its association with increased pathologic complete response following neoadjuvant chemotherapy have been shown^40^. Studies evaluating whether TILs can be used as a predictive biomarker of benefit to immunotherapy have also been performed. Exploratory analysis from KEYNOTE-086 demonstrated that patients who respond to pembrolizumab monotherapy have higher TILs compared to nonresponders^41^. In KEYNOTE-119, high TILs were significantly associated with better PFS and OS among patients treated in the pembrolizumab but not for those treated with chemotherapy^42^. More research on the utilization of TILs as a predictive biomarker to immunotherapy in breast cancer should be pursued.

In our present study, only 1/14 patients with targeted panel sequencing had a TMB > 10 mutations/Mb, which has been associated with longer PFS following immune checkpoint inhibitors in prior studies of mTNBC^43–45^. Additionally, six tumors had *PTEN* alterations, which have been associated with immunotherapy resistance in mTNBC and other tumors^43,46,47^. Altogether, these findings are consistent with prior work showing that mTNBC is immunologically “cold”, characterized by low TILs and reduced IFN-γ signature expression^13,14^.

Plasma proteomic studies further highlighted the profound level of immunosuppression in this study population. High plasma levels of immunosuppressive cytokines, chemokines, and immune checkpoints correlated with rapid progression. In addition, treatment was associated with increases in the hypoxia markers CA-IX, VEGF, and PlGF, which may indicate excessive vascular pruning. Finally, combination therapy was linked with substantial increases in plasma PD-1, which requires further investigation as a potential pharmacodynamic marker for this combination and potentially for PD-1 inhibitors alone.

Overall, the small sample sizes of the genomic sequencing cohorts and the low ORR prevented the evaluation of previously observed genomic correlates of response to immunotherapy in mTNBC, including TMB and *PTEN* alterations^43^. The single patient with targeted panel TMB > 10 in an earlier metastatic tumor also underwent subsequent whole-exome sequencing, which revealed TMB < 10 in the baseline, on-treatment, and post-treatment timepoints. This change in TMB may reflect tumor evolution or the tendency of targeted sequencing panels to overestimate TMB^48^. Furthermore, the one copy *PTEN* deletion in the baseline tumor biopsy of the patient with a durable partial response was not consistent with a previous study reporting that *PTEN* alterations were associated with worse response to checkpoint inhibitors in metastatic TNBC^43^. This discrepancy could be explained by the different sequencing methods, i.e., panel versus whole-exome sequencing, or the high immune pathway expression found in this tumor, which matches prior work showing improved immunotherapy responses in immune infiltrated mTNBC^42,49,50^.

The lack of benefit observed with this combination regimen may be explained by the safety and tolerability profile of cabozantinib in this population. Although this agent is currently approved for RCC and HCC at a dose of 60 mg oral daily, a previous trial from our group showed high rates of dose reduction in patients with breast cancer, and most patients were dose reduced to 40 mg^33^. The current trial therefore treated patients with the 40 mg dose of cabozantinib upfront. Even with this lower dose, the safety data remarkably showed that toxicities were frequent, leading to cabozantinib dose holds in all but one patient and dose reductions in half of patients. These dose modifications may have prevented cabozantinib from reaching adequate pharmacologic levels to exert a therapeutic effect. Therefore, other antiangiogenic agents may still have a synergistic role with immune checkpoint inhibitors in mTNBC.

This study has several limitations. First, patients were not selected according to PD-L1 status and most were previously treated for metastatic disease, whereas it is now recognized that intervention in earlier lines of treatment increases the likelihood of response to immune checkpoint inhibitors^51–53^. Second, only four patients had paired baseline and on-treatment biopsies, which precluded more robust analyses about the immunological consequences of this combination regimen. Finally, circulating biomarker data, while largely consistent with prior experience, remain hypothesis-generating and it should be considered as descriptive, as significance tests were not corrected for multiple hypotheses. Nevertheless, the finding related to the changes in circulating PD-1 is intriguing and warrants further validation as a potential pharmacodynamic marker of anti-PD-1-based therapies.

In conclusion, the combination of cabozantinib with nivolumab did not demonstrate clinical activity in this group of unselected patients with pretreated mTNBC. This is in contrast to the promising efficacy results seen with anti-VEGF/R and anti-PD-1/ L1 combination therapies in other cancers^20–23^. This discrepancy may in part be due to high rates of toxicity leading to frequent cabozantinib dose modifications. Molecular studies suggest a profound level of immunosuppression in these advanced mTNBC tumors and highlight the critical need to overcome immune resistance in this aggressive malignancy.

## Methods

### Study design and patient population

We conducted an open-label, single-arm, single center phase II study of cabozantinib with nivolumab in patients with mTNBC. Eligible patients had histologically or cytologically confirmed invasive breast cancer with metastatic disease that was measurable per RECIST 1.1^54^. Tumors were required to be estrogen receptor (ER)-negative and progesterone receptor-negative, defined as <10% expression by immunohistochemistry, and HER2-negative per the current American Society of Clinical Oncology/College of American Pathologists guidelines^55^. Research biopsies at baseline and on-treatment during days 15–28 of cycle 2 were mandatory for patients with safely accessible disease. Participants may have received 0–3 prior lines of chemotherapy for metastatic breast cancer. Additional inclusion criteria included age ≥18 years and Eastern Cooperative Oncology Group (ECOG) performance status ≤ 1. Exclusion criteria included prior treatment with a MET inhibitor or immune checkpoint inhibitor and evidence of cardiovascular or gastrointestinal tumor invasion.

### Study procedures

Nivolumab 480 mg was administered intravenously on day 1 of every 28-day cycle, and cabozantinib 40 mg was administered orally once daily for 28-day cycles. The first phase of the study included a safety run-in of the combination regimen. If ≥2 of the first six patients enrolled experienced a dose-limiting toxicity, the trial would have closed for further enrollment. Biopsies were performed at screening and during days 15–28 of cycle 2. Tissue from each biopsy was used for exploratory biomarker studies. In addition, research blood samples were collected from each patient at baseline, on day 1 of cycle 2 and each subsequent cycle, at each restaging visit, and once off treatment for progressive disease. All patients adhered to the protocol schedule for follow-up visits. The Dana-Farber Cancer Institute institutional review board approved the study (see protocol in Supplemental Materials), and written informed consent from all the trial participants was provided before study entry. The study was monitored by the Data Safety Monitoring Board of the Dana-Farber/Harvard Cancer Center.

### Statistical considerations

The primary endpoint was ORR according to RECIST 1.1. The study followed a Simon’s optimal two-stage design^56^. In the first stage, 18 patients were enrolled. If at least 3/18 patients achieved a complete or partial response, then an additional 17 patients would be enrolled. If at least 7/35 patients achieved a complete response or partial response, the combination regimen would be considered worthy of further study. Based on this design, a true ORR of 10% or less would not be of clinical interest and was the null hypothesis, while a true ORR of 30% would be considered a clinically meaningful level of response. The sample size had 90% power to declare the combination effective at this rate with a one-sided type I error rate of 5% under the null hypothesis. Secondary objectives included CBR, defined as the proportion of patients with a complete response, partial response or with stable disease at week 24, PFS, and adverse event frequency.

### Exploratory objectives

We explored the association of PD-L1 status, TILs, and tumor mutational burden (TMB) with outcomes. A total of 17 (94%) patients had PD-L1 testing assessed by the VENTANA assay using the SP142 antibody. PD-L1 positivity was defined as PD-L1–stained immune cells (IC) ≥ 1% of the tumor area^10^. Additionally, 16 (89%) patients had a hematoxylin and eosin stained section available to assess stromal TILs according to the International TILs Working Group guidelines^57^. Briefly, stromal TILs were quantified as the percentage of stroma within the invasive area covered by mononuclear cells over total intratumoral stromal area (0, 1, 5, 10, 15, 20, or >20 in 10% increments). All mononuclear cells, including lymphocytes and plasma cells, were scored, while granulocytes and polymorphonuclear leucocytes were excluded. The TIL level was evaluated as a continuous measure and as two ordinal levels (≤10% and >10%). TIL analysis was performed on archival or fresh tumor samples: 8 (53%) from primary tumors or local recurrences and 7 (43%) from metastatic tumors.

A total of 14 (78%) patients had genomic profiling using the next generation sequencing panel OncoPanel and had TMB assessed from these results. OncoPanel is run-in a Clinical Laboratory Improvement Amendments-certified laboratory environment and uses targeted exome sequencing to detect copy number alterations, single-nucleotide variants, and translocations across the full coding regions and selected intronic regions of a predefined subset of cancer-related genes using tumor-derived DNA^58,59^. The majority of patients (13) included in this study had testing done using OncoPanel version 3.1, which targets the full coding regions or selected intronic regions of 507 genes (exomic coverage region of 1.3 megabases [Mb])^59^. TMB was calculated as the number of nonsynonymous somatic mutations per megabase of exonic sequence data covered by the panel. High TMB was defined as ≥10 mutations per Mb and was used as a discrete variable in the analysis. OncoPanel was performed on archival tumor samples: 3 (21%) from primary tumors and 11 (79%) from metastatic tumors.

We also evaluated immunogenomic changes in the tumor immune microenvironment across the course of treatment. In paired tumor biopsies, whole-exome and transcriptome sequencing was performed on baseline and on-treatment samples from four patients, except for one on-treatment sample that contained insufficient DNA for whole-exome sequencing. In plasma samples, whole-exome sequencing was performed on circulating tumor DNA in baseline and post progression samples from three patients, as well as post progression samples from an additional two patients whose baseline plasma samples contained insufficient DNA for whole-exome sequencing. A total of three patients had both tissue and plasma whole-exome sequencing. Using the Olink platform (A probe—part # 84065, lot # A91713, exp 10/28/2021; B probe—part # 84066, lot # A91714, exp 10/28/2021), baseline and on-treatment plasma samples from all 18 patients were evaluated for 110 circulating angiogenic and inflammatory proteins and their correlation with response to combination therapy. Significance tests were not corrected for multiple hypotheses in this exploratory analysis of proteomic data.

Whole-exome sequencing was performed at the Broad Institute on seven RNAlater tumor samples and eight plasma samples using Illumina’s ICE hybrid-capture bait set as previously described^60–62^. Germline DNA was obtained from peripheral blood mononuclear cells. Exome sequencing data alignment and initial processing was performed using the Broad Institute Picard pipeline. BAM files uploaded into FireCloud (https://software.broadinstitute.org/firecloud/). Sequencing data were passed through additional quality-control and processing methods in FireCloud. Quality-control cutoffs were mean target coverage > 50× (tumor) and >20× (matched normal; GATK Depth of Coverage^63^), cross-contamination of samples estimation (ContEst^64^) < 5%, tumor purity (ABSOLUTE^65^, FACETS^66^) ≥ 9%, and tumor-in-normal contamination (deTIN^67^) < 30%.

We used an adaptation of the Getz Lab Cancer Genome Analysis WES pipeline (https://docs.google.com/document/d/1VO2kX_fgfUd0x3mBS9NjLUWGZu794WbTepBel3cBg08) developed at the Broad Institute to call, filter and annotate somatic mutations with modifications to enhance variant classification. For variant calling, the MuTect method^68^ was employed to identify somatic single-nucleotide variants with computational filtering of artifacts introduced by DNA fixation procedures^63^ and DNA oxidation during sequencing^69^. Strelka was used to identify small insertions or deletions^70^, and panel of normal filtering was utilized for rare artifacts specific to the bait set used^68^. Oncotator was applied to annotate identified alterations^71^.

Only nonsynonymous mutations (i.e., missense, nonsense, indel, and splice site) were included to enrich for functional genomic effects. Tumor mutation burden was defined as the nonsynonymous mutational burden normalized by megabases covered at adequate depth to detect variants with 80% power using MuTect given estimated tumor purity by ABSOLUTE^61^. The number of bases covered at a given depth threshold in the tumor was determined using the GATK DepthOfCoverage method^63^. Tumor purity and ploidy were estimated using ABSOLUTE^65^ and FACETS^66^. Clustering and clonal evolutionary analyses on samples from the same patient were performed with PhylogicNDT^72^.

The total number of copy number alterations for each tumor was calculated using an adapted binary segmentation method (CapSeg)^73^, and genes were annotated with Oncotator^71^. Allelic copy number alterations were identified by incorporating heterozygous single-nucleotide polymorphisms into the binary segmentation method (Allelic CapSeg). Allelic segments were then adjusted for tumor purity and ploidy. Allelic amplifications and deletions were then called with previously described methods integrating segment focality and the purity- and ploidy-corrected allelic copy number^61,74^.

Whole-transcriptome sequencing was performed at the Broad Institute on paired baseline and on-treatment RNAlater tumor samples from four patients using established methods^60,62^. RNA sequencing results were aligned using STAR and then quantified with RSEM to yield gene-level expression in transcripts per million (TPM)^75,76^. Samples were clustered across quality-control metrics using principal-component analysis, which revealed no outlier samples.

For whole-transcriptome sequencing analyses, differential gene pathway expression was evaluated with geneset enrichment analysis using the cancer hallmark genesets from the Molecular Signatures Database (MSigDB; https://www.gsea-msigdb.org/gsea/msigdb/collections.jsp)^77^, and tumor immune cell composition was assessed with the immune deconvolution algorithm CIBERSORTx using the LM22 signature matrix^78^. Tumor-infiltrating lymphocytes were defined as T cells, NK cells, B cells, and plasma cells identified by CIBERSORTx^78^. The false discovery rate (FDR) for geneset enrichment analyses was controlled with the Benjamini–Hochberg method with a threshold of q < 0.05. Additional formal statistical tests for significance were not performed given the power limitations of the small sample numbers in each cohort. Statistical analyses were run-in R studio version 1.2.5001.

### Reporting summary

Further information on research design is available in the Nature Research Reporting Summary linked to this article.

## Supplementary information

Supplementary Data 1

Supplementary Information

Reporting Summary

## Data Availability

The data generated and analysed during this study are described in the following data record: 10.6084/m9.figshare.14578365^79^. The whole-exome and transcriptome data have been deposited in the dbGaP repository under accession https://identifiers.org/dbgap:phs002419.v1.p1^80^. In order to protect patient privacy, these data are controlled access. Details of how to request access can be found on the dbGaP landing page. The genomic profiling data are in Supplementary Tables 2 and 4. The supplementary tables are also available in.xlsx format as part of the figshare data record.

## Code availability

All software and bioinformatic tools used in the present study are publicly available from the indicated references below and in the Methods section. Software used: Getz Lab Cancer Genome Analysis WES pipeline (https://docs.google.com/document/d/1VO2kX_fgfUd0x3mBS9NjLUWGZu794WbTepBel3cBg08); Mutect (v1.1.6); Strelka (v.1.0.11; ReCapSeg (https://gatkforums.broadinstitute.org/gatk/categories/recapseg); ABSOLUTE (https://software.broadinstitute.org/cancer/cga/absolute); ONCOTATOR (https://software.broadinstitute.org/cancer/cga/ oncotator); PhylogicNDT (https://github.com/broadinstitute/PhylogicNDT); STAR(v2.7.0); RSEM(v1.3.1) R(v4.0.2)-packages: pheatmap(v1.0.12), ggplot(v3.0.4), ggplot2(v3.3.2)Websites: CIBERSORTx (http://cibersortx.stanford.edu); GSEA (https://cloud.genepattern.org).
