# Supplementary material for: Nivolumab in combination with cabozantinib for metastatic triple-negative breast cancer: a phase II and biomarker study
Source: NPJ Breast Cancer. 2021 Aug 25;7:110. doi: 10.1038/s41523-021-00287-9 (PMC8387440; doi:10.1038/s41523-021-00287-9)
Supplement: Supplementary file 2 — Supplementary Information [file 41523_2021_287_MOESM2_ESM.pdf]

## Supplementary Online Content

**Supplementary Figure 1.** Targeted panel co-mutation plot of breast cancer genes

**Supplementary Figure 2.** Kaplan-Meier curves for progression-free survival and overall survival by *PTEN* and *PIK3CA* alterations

**Supplementary Figure 3.** Targeted panel co-mutation plot of immunotherapy-related genes

**Supplementary Figure 4.** Whole exome sequencing co-mutation plot of immunotherapy-related genes

**Supplementary Figure 5.** Tumor clonal evolution of durable responder

**Supplementary Figure 6.** Immune cell deconvolution of RNA sequencing data

**Supplementary Figure 7.** Change in immune cells inferred by RNA sequencing

**Supplementary Table 1.** Dose modification

**Supplementary Table 2.** Immune gene sets enriched in responders in baseline biopsies

**Supplementary Table 3.** Immune gene sets enriched in responders in on-treatment biopsies

**Supplementary Table 4.** Immune gene sets enriched in on-treatment biopsies

**Supplementary References**

**Supplementary Data 1 file (excel)**

-Supplementary Figure 1 Data

-Supplementary Figure 2 Data

-Supplementary Figure 3 Data

-Figure 3 Data

-Supplementary Figure 4 Data

-Supplementary Figure 5 Data

-Transcript Per Million Gene Expression Data for Figure 3B, Supplementary Tables 2-4, and Supplementary Figures 6-7

-Plasma biomarker association with progression free survival after cabozantinib plus nivolumab treatment in mTNBC patients

-Association between plasma biomarker change at after one cycle of cabozantinib plus nivolumab treatment and progression free survival in mTNBC patients.

**Supplementary Figure 1. Targeted panel co-mutation plot of breast cancer genes** Targeted panel sequencing breast cancer gene alterations in archival primary or metastatic tumors from 14 patients showed no clear association with response: each column represents a patient ordered from longest to shortest progression-free survival (PFS).

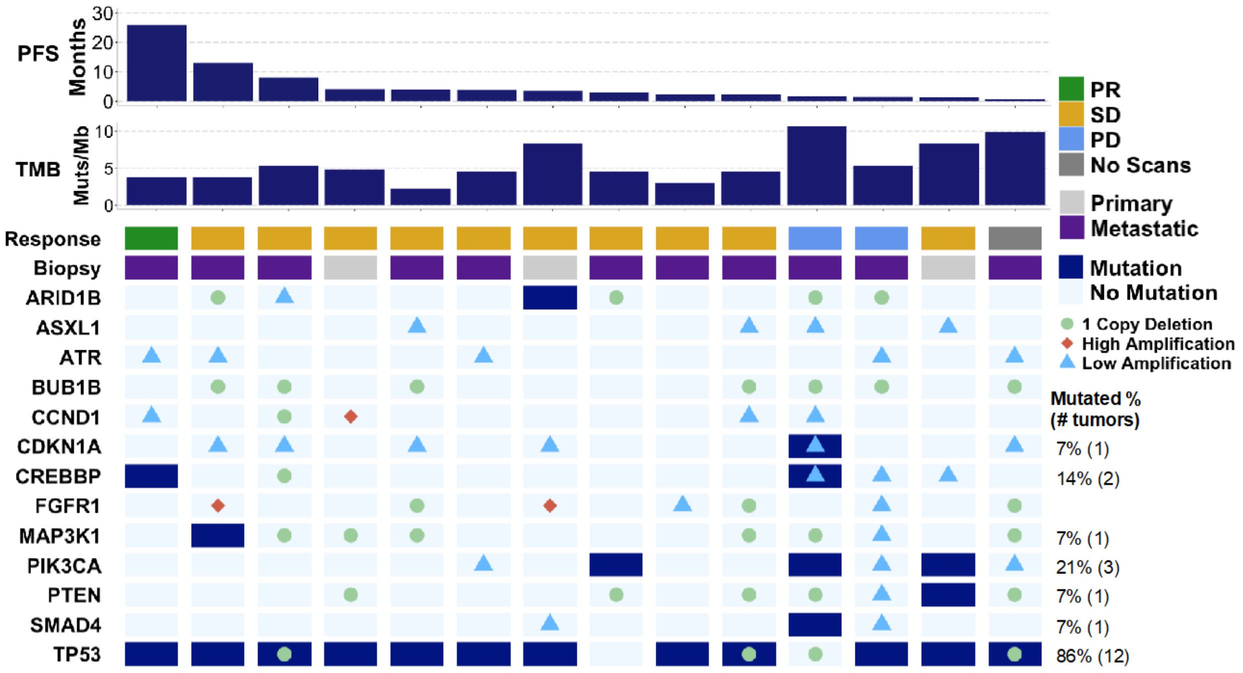

**Supplementary Figure 2. Kaplan-Meier curves for progression-free survival and overall survival by *PTEN* and *PIK3CA* alterations** (a) Progression-free survival and (b) overall survival by *PTEN* alteration (absent vs. present); (c) Progression-free survival and (d) overall survival by *PIK3CA* alteration (absent vs. present). WT: wild type.

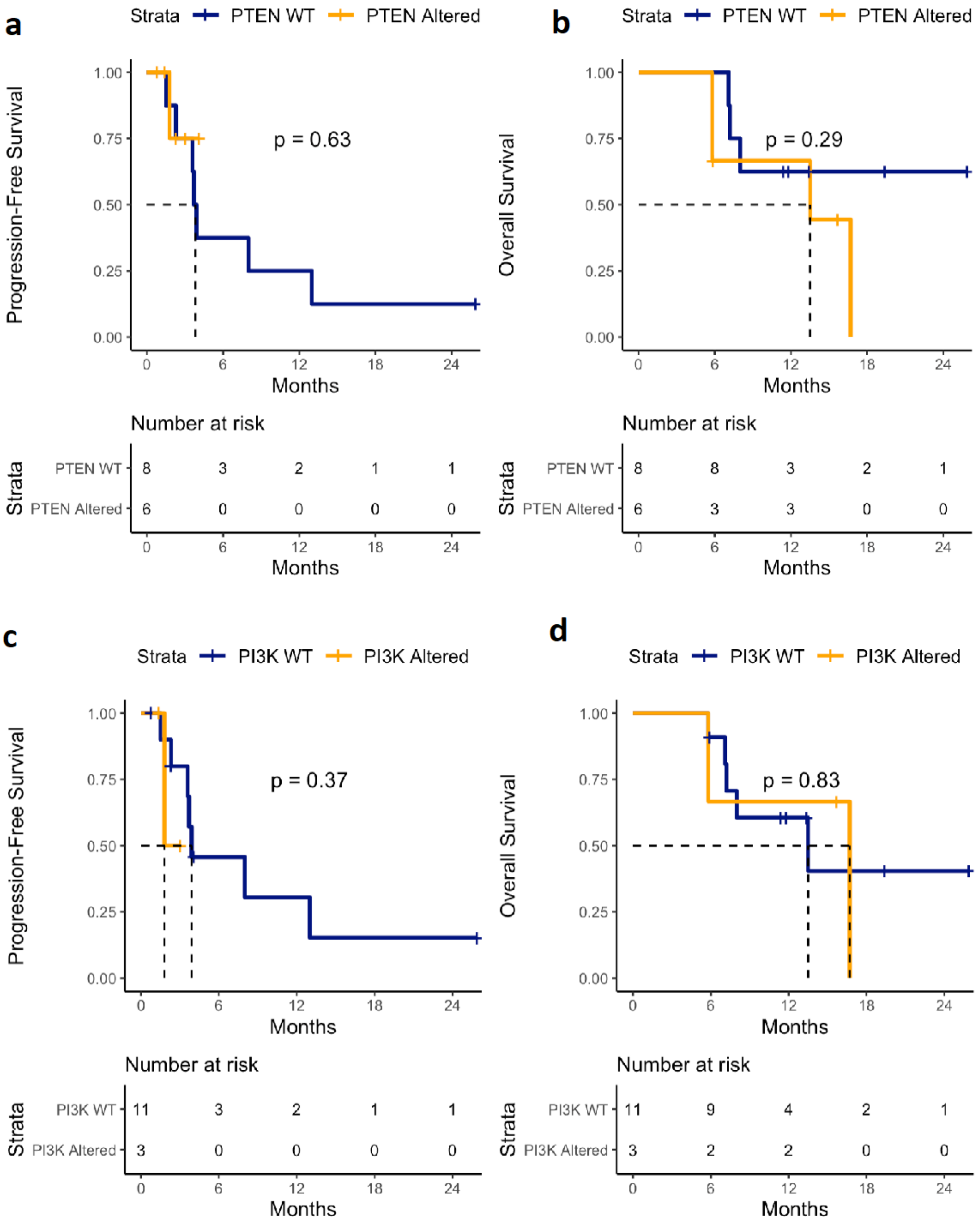

**Supplementary Figure 3. Targeted panel co-mutation plot of immunotherapy-related genes**

Immunotherapy gene alterations in archival primary or metastatic tumor biopsies from 14 patients with targeted panel sequencing showed no clear association with response: each column represents a patient ordered from longest to shortest progression-free survival (PFS).

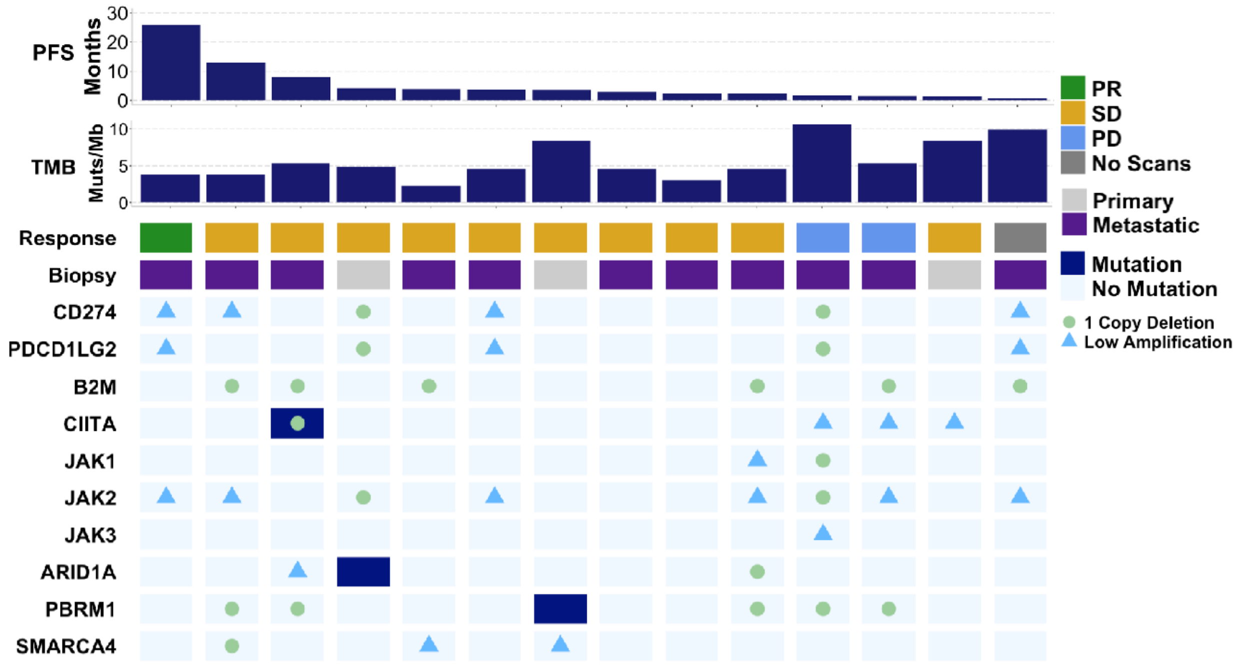

**Supplementary Figure 4. Whole exome sequencing co-mutation plot of immunotherapy-related genes** Immunotherapy gene alterations in baseline, on treatment, and post treatment tissue and blood biopsies from 6 patients with whole exome sequencing showed no clear association with response: each column represents a tumor or blood biopsy with samples grouped by patient ordered from longest to shortest progression-free survival.

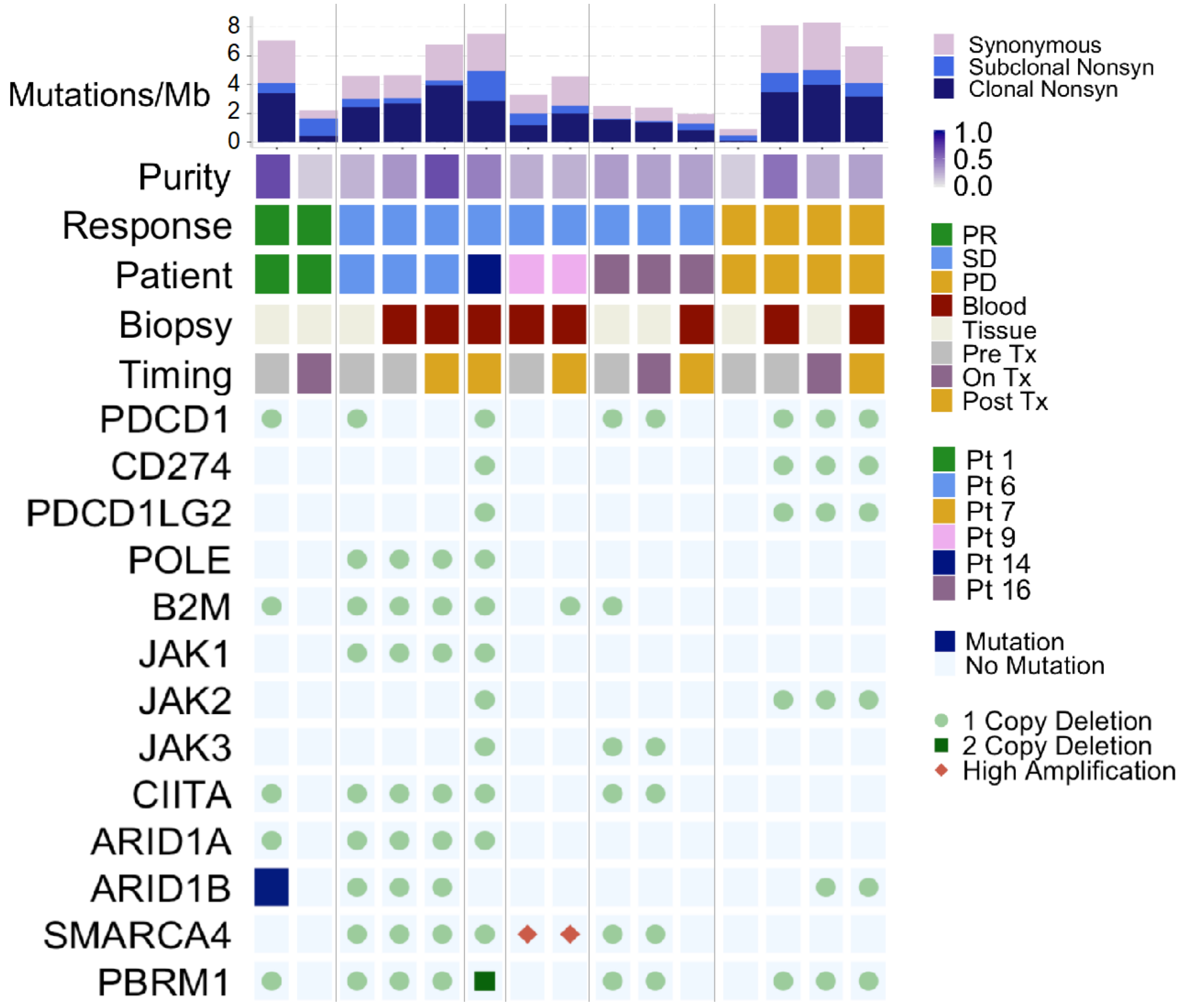

**Supplementary Figure 5. Tumor clonal evolution of durable responder** Mutation clustering analyses with PhylogicNDT indicated that the cancer cell fraction of canonical breast cancer gene mutations, including TP53, ARID1B, and FGFR3, decreased from baseline to on-treatment tissue biopsy timepoints in the patient with a durable partial response.

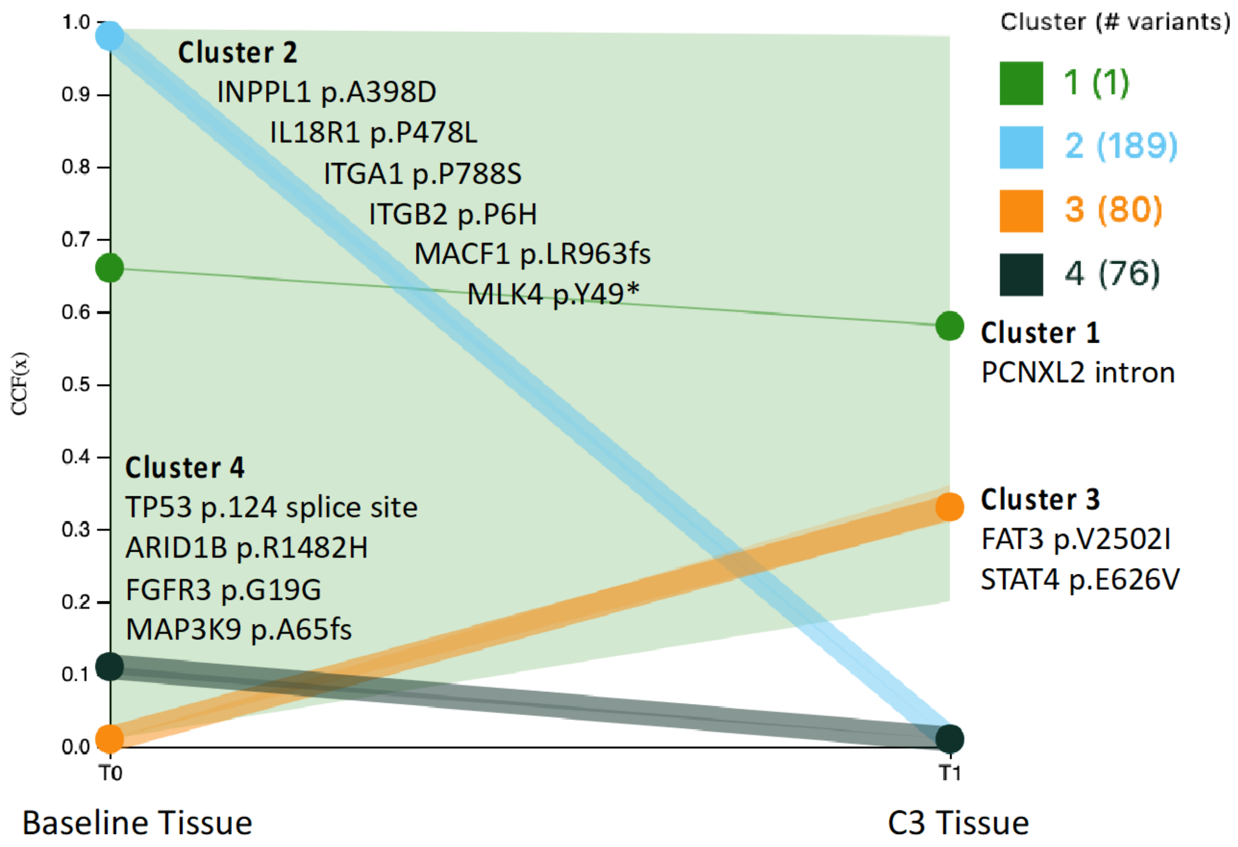

**Supplementary Figure 6. Immune cell deconvolution of RNA sequencing data** Immune cell deconvolution showed no difference in tumor infiltrating lymphocytes (TILs) by response group at baseline (a) or on treatment (b), as well as no difference in absolute immune infiltrate at baseline (c) or on treatment (d). Responders (green) consisted of 1 patient with a durable partial response and 1 patient with stable disease > 6 months, while non-responders (yellow) included 1 patient with progressive disease and 1 patient with stable disease < 6 months.

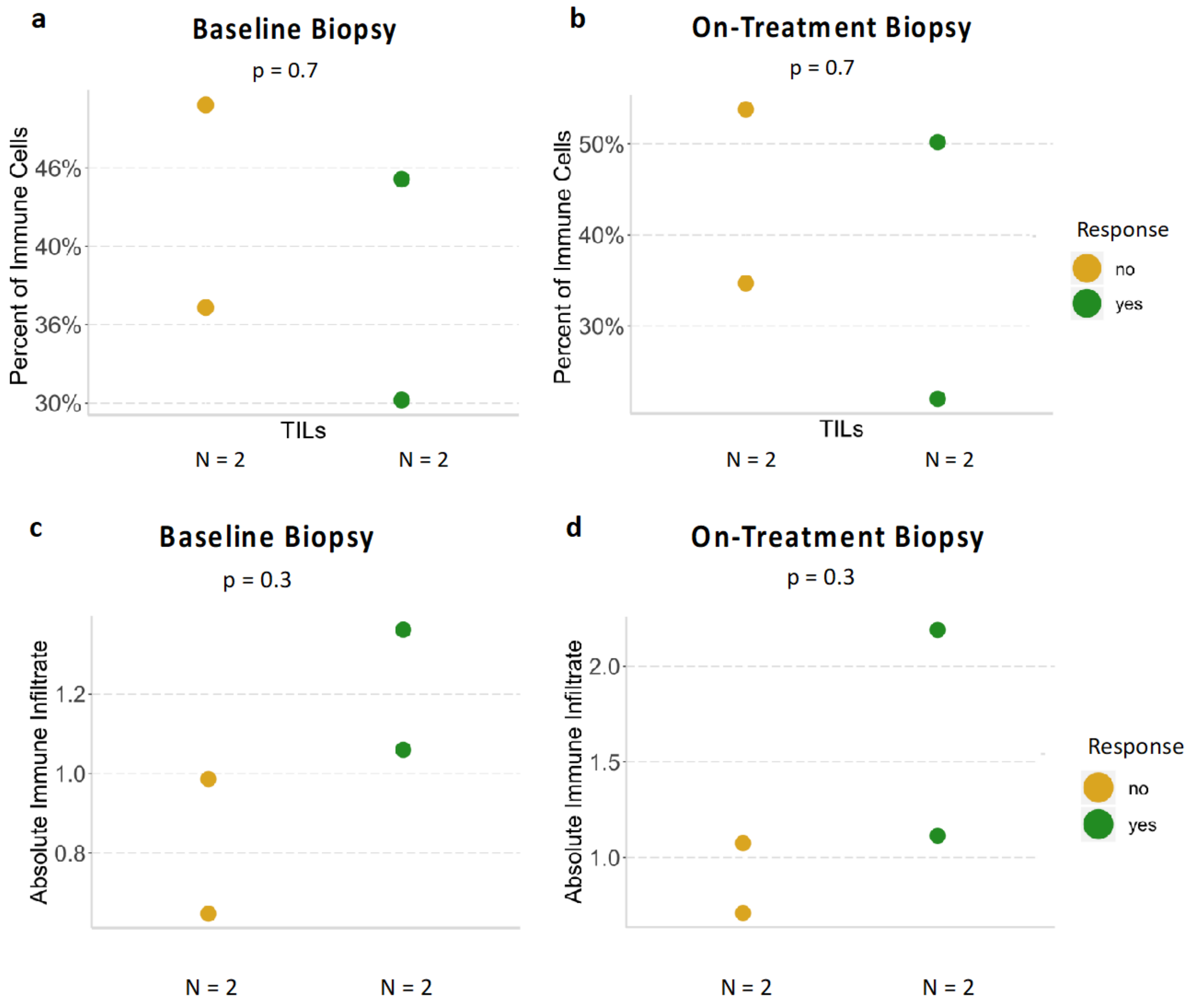

**Supplementary Figure 7. Change in immune cells inferred by RNA sequencing** Immune cell deconvolution showed no consistent change with treatment in tumor infiltrating lymphocytes (TILs) in responders (a) or non-responders (b), as well as no consistent change with treatment in absolute immune infiltrate in responders (c) or non-responders (d). Responders (green) consisted of 1 patient with a durable partial response and 1 patient with stable disease > 6 months, while non-responders (yellow) included 1 patient with progressive disease and 1 patient with stable disease < 6 months.

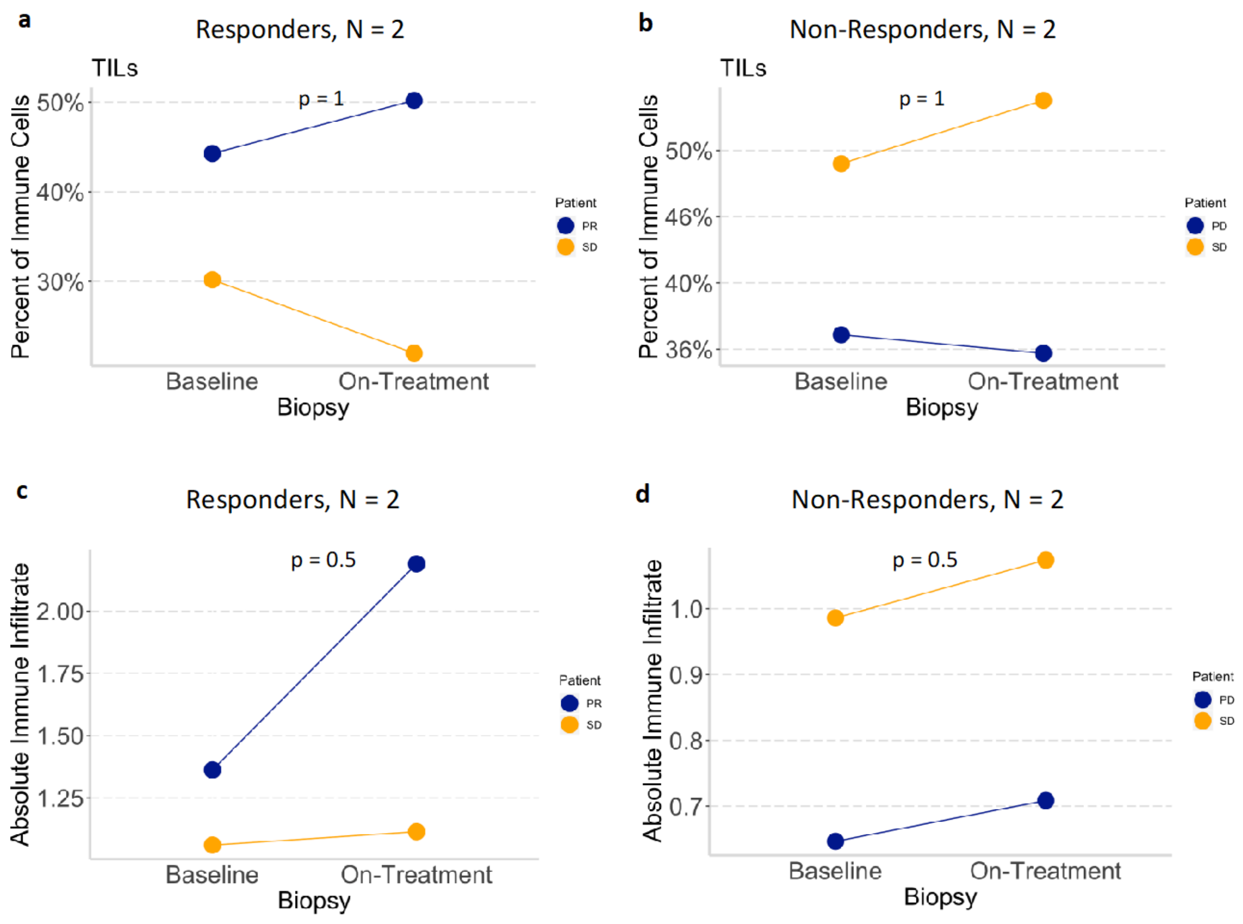

**Supplementary Table 1. Dose modification**

| | Cabozantinib | Nivolumab |
|---|---|---|
| Dose held, n (%) | 17 (94.4) | 5 (27.8) |
| Dose reduced, n (%) | 9 (50) | NA |
| Dose discontinuation due to toxicity, n (%) | 5 (27.8) | |

**Supplementary Table 2. Immune gene sets enriched in responders in baseline biopsies**

| Rank | MSigDb Hallmark Gene Set | NES | FDR q-val |
|---|---|---|---|
| 1 | HALLMARK_ALLOGRAFT_REJECTION | 2.49 | 0.000 |
| 2 | HALLMARK_TNFA_SIGNALING_VIA_NFKB | 1.99 | 0.000 |
| 3 | HALLMARK_INFLAMMATORY_RESPONSE | 1.97 | 0.000 |
| 4 | HALLMARK_IL6_JAK_STAT3_SIGNALING | 1.89 | 0.000 |
| 5 | HALLMARK_INTERFERON_GAMMA_RESPONSE | 1.54 | 0.042 |

**Supplementary Table 3. Immune gene sets enriched in responders in on-treatment biopsies**

| Rank | MSigDb Hallmark Gene Set | NES | FDR q-val |
|---|---|---|---|
| 1 | HALLMARK_EPITHELIAL_MESENCHYMAL_TRANSITION | 2.36 | 0.000 |
| 2 | **HALLMARK_ALLOGRAFT_REJECTION** | 2.27 | 0.000 |
| 3 | **HALLMARK_TNFA_SIGNALING_VIA_NFKB** | 2.24 | 0.000 |
| 4 | HALLMARK_MYOGENESIS | 2.01 | 0.000 |
| 5 | HALLMARK_HYPOXIA | 1.97 | 0.000 |
| 6 | **HALLMARK_INFLAMMATORY_RESPONSE** | 1.80 | 0.001 |
| 7 | **HALLMARK_IL6_JAK_STAT3_SIGNALING** | 1.78 | 0.001 |

**Supplementary Table 4. Immune gene sets enriched in on-treatment biopsies**

| Rank | MSigDb Hallmark Gene Set | NES | FDR q-val |
|---|---|---|---|
| 1 | HALLMARK_EPITHELIAL_MESENCHYMAL_TRANSITION | -2.50 | 0.000 |
| 2 | **HALLMARK_TNFA_SIGNALING_VIA_NFKB** | -2.45 | 0.000 |
| 3 | HALLMARK_HYPOXIA | -2.28 | 0.000 |
| 4 | **HALLMARK_ALLOGRAFT_REJECTION** | -2.11 | 0.000 |
| 5 | **HALLMARK_INFLAMMATORY_RESPONSE** | -2.05 | 0.000 |
| 6 | HALLMARK_COMPLEMENT | -2.00 | 0.000 |
| 7 | HALLMARK_APOPTOSIS | -1.96 | 0.000 |
| 8 | HALLMARK_P53_PATHWAY | -1.77 | 0.002 |
| 9 | HALLMARK_KRAS_SIGNALING_UP | -1.75 | 0.002 |
| 10 | **HALLMARK_IL6_JAK_STAT3_SIGNALING** | -1.72 | 0.003 |
| 11 | **HALLMARK_INTERFERON_GAMMA_RESPONSE** | -1.71 | 0.003 |
| 12 | HALLMARK_MTORC1_SIGNALING | -1.69 | 0.003 |

FDR, false discovery rate; MSigDb, Molecular Signatures Database;[1] NES, normalized enrichment score.

**Supplementary References**


1 Liberzon, A. *et al.* The Molecular Signatures Database (MSigDB) hallmark gene set collection. *Cell Syst* **1**, 417-425, doi:10.1016/j.cels.2015.12.004 (2015).
