# Supplementary material for: Nivolumab in combination with cabozantinib for metastatic triple-negative breast cancer: a phase II and biomarker study
Source: NPJ Breast Cancer. 2021 Aug 25;7:110. doi: 10.1038/s41523-021-00287-9 (PMC8387440; doi:10.1038/s41523-021-00287-9)
Supplement: Supplementary file 3 — Reporting Summary [file 41523_2021_287_MOESM3_ESM.pdf]

nature research

Corresponding author(s): Sara Tolaney
Last updated by author(s): Oct 27, 2020

Reporting Summary

Nature Research wishes to improve the reproducibility of the work that we publish. This form provides structure for consistency and transparency in reporting. For further information on Nature Research policies, see our Editorial Policies and the Editorial Policy Checklist.

Statistics

For all statistical analyses, confirm that the following items are present in the figure legend, table legend, main text, or Methods section.

n/a	Confirmed	
☐	☒	The exact sample size (*n*) for each experimental group/condition, given as a discrete number and unit of measurement
☒	☐	A statement on whether measurements were taken from distinct samples or whether the same sample was measured repeatedly
☐	☒	The statistical test(s) used AND whether they are one- or two-sided<br>*Only common tests should be described solely by name; describe more complex techniques in the Methods section.*
☐	☒	A description of all covariates tested
☐	☒	A description of any assumptions or corrections, such as tests of normality and adjustment for multiple comparisons
☐	☒	A full description of the statistical parameters including central tendency (e.g. means) or other basic estimates (e.g. regression coefficient) AND variation (e.g. standard deviation) or associated estimates of uncertainty (e.g. confidence intervals)
☐	☒	For null hypothesis testing, the test statistic (e.g. *F*, *t*, *r*) with confidence intervals, effect sizes, degrees of freedom and *P* value noted<br>*Give P values as exact values whenever suitable.*
☒	☐	For Bayesian analysis, information on the choice of priors and Markov chain Monte Carlo settings
☒	☐	For hierarchical and complex designs, identification of the appropriate level for tests and full reporting of outcomes
☐	☒	Estimates of effect sizes (e.g. Cohen's *d*, Pearson's *r*), indicating how they were calculated

Our web collection on statistics for biologists contains articles on many of the points above.

Software and code

Policy information about availability of computer code

Data collection	All data needed to regenerate figures and results are provided in supplemental information. Raw sequencing data (BAMs) can be made available to any reader directly upon reasonable request.
Data analysis	All analytical techniques are described and cited in the Methods section. Results generated using publically available websites/tools with parameters are described in Methods. Code to regenerate figures will be available in Github.<br>Software used: Mutect (v1.1.6); Strelka (v.1.0.11; ReCapSeg (https://gatkforums.broadinstitute.org/gatk/categories/recapseg); ABSOLUTE (https://software.broadinstitute.org/cancer/cga/absolute); ONCOTATOR (https://software.broadinstitute.org/cancer/cga/ oncotator); PhylogicNDT (https://github.com/broadinstitute/PhylogicNDT); STAR(v2.7.0); RSEM(v1.3.1)<br>R(v4.0.2)-packages: pheatmap(v1.0.12), ggplot(v3.0.4), ggplot2(v3.3.2)<br>Websites: CIBERSORTx (http://cibersortx.stanford.edu); GSEA (https://cloud.genepattern.org)

For manuscripts utilizing custom algorithms or software that are central to the research but not yet described in published literature, software must be made available to editors and reviewers. We strongly encourage code deposition in a community repository (e.g. GitHub). See the Nature Research guidelines for submitting code & software for further information.

Data

Policy information about availability of data

All manuscripts must include a data availability statement. This statement should provide the following information, where applicable:

- Accession codes, unique identifiers, or web links for publicly available datasets
- A list of figures that have associated raw data
- A description of any restrictions on data availability

A data availability statement is provided, and data for figures are provided in supplemental information

Field-specific reporting

Please select the one below that is the best fit for your research. If you are not sure, read the appropriate sections before making your selection.

☒ Life sciences ☐ Behavioural & social sciences ☐ Ecological, evolutionary & environmental sciences

For a reference copy of the document with all sections, see nature.com/documents/nr-reporting-summary-flat.pdf

Life sciences study design

All studies must disclose on these points even when the disclosure is negative.

Sample size	18
Data exclusions	There was no data exclusion
Replication	Not aplicable
Randomization	This was a non-randomized clinical trial
Blinding	This was a single arm study, thus there was no blinding.

Reporting for specific materials, systems and methods

We require information from authors about some types of materials, experimental systems and methods used in many studies. Here, indicate whether each material, system or method listed is relevant to your study. If you are not sure if a list item applies to your research, read the appropriate section before selecting a response.

Materials & experimental systems

n/a	Involved in the study	
☒	☐	Antibodies
☒	☐	Eukaryotic cell lines
☒	☐	Palaeontology and archaeology
☒	☐	Animals and other organisms
☐	☒	Human research participants
☐	☒	Clinical data
☒	☐	Dual use research of concern

Methods

n/a	Involved in the study	
☒	☐	ChIP-seq
☒	☐	Flow cytometry
☒	☐	MRI-based neuroimaging

Human research participants

Policy information about studies involving human research participants

Population characteristics	Eligible patients had histologically or cytologically confirmed invasive breast cancer with metastatic disease that was measurable per RECIST 1.1 . Tumors were required to be estrogen receptor (ER)-negative and progesterone receptor-negative, defined as < 10% expression by immunohistochemistry, and HER2-negative per the current American Society of Clinical Oncology/College of American Pathologists guidelines
Recruitment	Patients were recruited during clinical practice.
Ethics oversight	The Dana-Farber Cancer Institute institutional review board approved the study.

Note that full information on the approval of the study protocol must also be provided in the manuscript.

Clinical data

Policy information about clinical studies

All manuscripts should comply with the ICMJE guidelines for publication of clinical research and a completed CONSORT checklist must be included with all submissions.

Clinical trial registration	NCT03316586
Study protocol	The study protocol can is attached with the manuscript submission and will be available in the supplementary material
Data collection	From 12/15/2017 to 1/24/2019, 18 patients were enrolled into the trial at Dana-Faber Cancer Institute..

Outcomes

The primary endpoint was objective response rate according to RECIST 1.1. Secondary objectives included clinical benefit rate (CBR), defined as the proportion of patients with a complete response, partial response or with stable disease at week 24, progression-free survival, and adverse event frequency.
